# The Treatment of Pulpless Teeth

**Published:** 1888-10

**Authors:** George F. Cheney

**Affiliations:** St. Johnsburg, Vt.


### THE TREATMENT OF PULPLESS TEETH.

BY GEO. F. CHENEY, ST. JOHNSBURG, VT.

Read before the Connecticut Valley and Massachusetts Societies at their Union Meeting, Boston, July 10, 1888.

Since the reading of Dr. Stockwell's paper before the Vermont State Dental Society in March, 1886, published in the May number of the Archives of Dentistry, upon "The Treatment and Filling of Root Canals at a Single Sitting," I have given the subject considerable thought and study.

The treatment I have followed is not in strict accordance with Dr. Stockwell, but is taken from the ideas advanced by him and several other writers.

The treatment of pulpless teeth is and has been for some time past receiving a good deal of attention at all society meetings, and to my mind is a subject that cannot be too fully discussed, and is one we should be constantly studying, it being one of the most important we have to deal with. While I do not advocate the treatment and filling of all classes of these teeth at a single sitting, I do claim that a majority of the teeth with freshly devitalized pulps, and those in which the pulps have recently become putrescent, had better be filled at the first sitting than to prolong the treatment. But with teeth where there has been much periosteal trouble, or where the pulps have long been septic, it is insufficient, and I think an unsafe practice to introduce a disinfectant, immediately following it with a germicide, and that by a permanent root filling.

Frequently we find the tissues, not only of the tooth itself, but of the territory beyond infected, and impossible to be made entirely aseptic at a single sitting. Again, we frequently find teeth where there is an effusion of moisture of one character or another which is impossible to check at once; with teeth of this class the treatment should be prolonged, but very rarely beyond the second sitting.

Pulpless teeth, although sometimes called such, are not necessarily dead teeth. The destruction of the pulp simply deprives the dentine of it chief or perhaps only supply of nourishment. It does not at all effect the connection of the tooth with the jaw or the nourishment of the cementum. The connection of the tooth with the jaw is sustained by the peridental membrane, that being abundantly supplied with blood by arteries coming to it at the apical space, and through the alveolar walls. While we may have an abscess in the apical space that destroys the blood supply from that source, the membrane is still well supplied through the alveolar wall.

Therefore, by properly protecting the dentine, and by careful treatment a tooth that has been deprived of its pulp, it can be put in condition to be maintained for years or perhaps a lifetime.

We have been taught that micro-organisms are an important factor in, if not the cause of the formation of pus; that the saliva is one of the best culture mediums known for these organisms, and that they are nearly always present in it. It therefore follows that we should use extra care to exclude the saliva from a tooth while under treatment. Under all circumstances, when opening into a pulp canal, whether to extirpate the pulp or to change a dressing, adjust the rubber dam and disinfect the teeth included, and all instruments used. It is the only way to insure thorough-

2

ness, and thoroughness is pretty sure to give success in all cases.

The treatment of teeth with newly devitalized pulps is very simple. Remove all decay and septic matter from the cavity, wash the tooth cavity and rubber dam thoroughly with one one-thousandth solution of bi-chloride of mercury, open the pulp-chamber in a way that will give free access to the root canals, and with a freshly disinfected broach remove the pulp, being careful to leave no part of it; wash canal with the bi-chloride of mercury solution, and dry thoroughly, using cones made of bibulous paper, and hot air. Now the canal is ready to fill, and if the work has been carefully done abscess cannot occur.

For filling root canals, anything that will hermetrically seal the apex is allowable, although gutta-percha is in most general use, and I think it the best material on account of the ease with which it can be introduced. Under no consideration fall into the fatal error of using cotton; it will not seal the apex, and disaster will sooner or later follow. I fill these teeth by first pumping a little gutta-percha and chloroform of about the consistency of cream to the apex, following with a cone, working it well into the canal, until the patient gives a little jerk, which proves the apex has been reached.

When the canals are very small; for instance, mesial roots of lower and buccal roots of superior molars, after the gutta-percha has been worked in as much as possible, introduce a piece of fine gold wire, being careful not to crowd it through the apex, it will force the gutta-percha more solidly into the root.

Teeth with putrescent pulps are frequently found where the pulp has died from exposure or some other cause. Such cases very often occur under oxyphosphate fillings. The treatment is very simple. Remove all decay and septic matter from the cavity and canal, using care not to force anything through the apex; then, with a syringe and hydrogen peroxide, cleanse the canal thoroughly. This cleansing should be continued until all bubling ceases, after which wipe the canal dry and introduce into it the bichloride of mercury solution, allowing it to remain a few minutes; dry thoroughly with hot air, and the root is ready to fill.

When the pulp has been dead and putrescent for a long time, or where there has been apical trouble or blind abscess, the treatment is more complicated. These cases I never fill at the first sitting, but disinfect as thoroughly as possible, and fill the root with cotton saturated with iodoform and eucalyptol, sealing the cavity with gutta-percha, dismissing the patient for about a week, with instructions to return at the first indication of trouble. If at the end of this time I find no signs of pus, I fill; if pus is found, repeat the treatment, dismissing the patient for another weeks. It very seldom becomes necessary to prolong the treatment beyond this. If, after the tooth is filled permanently, there should be any serious trouble, resort to depletion, cutting deep down at the apex and toward, but not to the margin of the gum. This relieves the tension, and has always met the requirements of the case, so far as my experience has been.

I have had one or two cases of blind abscess where, after repeating the treatment several times, I still found a slight watery discharge, tinged with blood. This I arrested with alcohol and hot air, then filled, having no after trouble.

Cases of acute alveolar abscess as a rule require very little treatment. Open into the pulp canal, disinfect as much as possible with hydrogen peroxide, leaving the canal open for a day; then continue treatment. When opening into the canal, if the tooth is very sore, the operation can be made less painful by tying a string about the tooth, allowing the patient to pull down on it.

If the tooth should be too sore to admit the opening of the pulp chamber, make an opening from the outside with a lance. This operation can be made comparatively painless by drying the gum and marking it with carbolic acid crystals. This opening will give the pus a chance to escape. When after a day the inflammation will have subsided sufficiently for the opening of the pulp chamber and the continuance of the treatment, which is about the same as given for teeth with putrescent pulps, filling temporarily for a week. Usually the two sittings are all that is required.

For chronic alveolar abscess the treatment is nearly the same as heretofore given. After opening and cleansing the pulp chambers and canal as before, introduce the hydrogen peroxide, allowing it to pass through the apex and fistula; then pump the one one-thousandth solution of bichloride of mercury into the canal, allowing it also to pass through the apex and fistula. I sometimes use a stronger solution of the bichloride of mercury—say one grain to the half-ounce—for the roots; but not to force through the apex.

I fill these teeth temporarily until the fistula commences to heal. The fistula will, in most cases, close up in a few days without further treatment; although we sometimes find necrosed bone, which should be removed with a sharp burr, after enlarging the fistulous opening. Sometimes, when I have found fistulæ that were obstinate about healing, tincture of iodine introduced along the track has produced the desired effect and brought about healthy granulations.

Fistulous openings at the margin of the gum I have found the most difficult to heal. I think it is in these cases we are most likely to find necrosed bone.

If, at the time I remove the temporary filling from any tooth under treatment, signs of pus are found, I repeat the treatment, dismissing the patient for another week.

Dr. J. A. Dunn, of Chicago, has invented a syringe that I have found very useful for treating teeth, and especially convenient for introducing medicaments through the fistula to the apex of a tooth.

In writing this paper I have attempted nothing scientific; but to give a plan of treatment that, if carried out with care and thoroughness, will give satisfactory results, and of starting the discussion of a subject that is to me very important, and one upon which I wish to become more thoroughly informed.

